# Cross-Reactivity and Sequence Homology Between Alpha-Synuclein and Food Products: A Step Further for Parkinson’s Disease Synucleinopathy

**DOI:** 10.3390/cells10051111

**Published:** 2021-05-05

**Authors:** Aristo Vojdani, Aaron Lerner, Elroy Vojdani

**Affiliations:** 1 Immunosciences Laboratory, Inc., Los Angeles, CA 90035, USA; drari@msn.com; 2 Cyrex Laboratories, Phoenix, AZ 85034, USA; 3 Department of Preventive Medicine, Loma Linda University School of Medicine, Loma Linda, CA 92354, USA; 4 Chaim Sheba Medical Center, Zabludowicz Center for Autoimmune Diseases, Tel-Hashomer 52621, Israel; 5 Regenera Medical,11620 Wilshire Blvd., Ste. 470, Los Angeles, CA 90025, USA; evojdani@gmail.com

**Keywords:** Parkinson’s disease, α-synuclein, antibodies, antigen, food, cross-reactivity, sequence homology, synucleinopathy, BLAST, gut-brain axis

## Abstract

Introduction: Parkinson’s disease is characterized by non-motor/motor dysfunction midbrain neuronal death and α-synuclein deposits. The accepted hypothesis is that unknown environmental factors induce α-synuclein accumulation in the brain via the enteric nervous system. Material and Methods: Monoclonal antibodies made against recombinant α-synuclein protein or α-synuclein epitope 118–123 were applied to the antigens of 180 frequently consumed food products. The specificity of those antibody-antigen reactions was confirmed by serial dilution and inhibition studies. The Basic Local Alignment Search Tool sequence matching program was used for sequence homologies. Results: While the antibody made against recombinant α-synuclein reacted significantly with 86/180 specific food antigens, the antibody made against α-synuclein epitope 118–123 reacted with only 32/180 tested food antigens. The food proteins with the greatest number of peptides that matched with α-synuclein were yeast, soybean, latex hevein, wheat germ agglutinin, potato, peanut, bean agglutinin, pea lectin, shrimp, bromelain, and lentil lectin. **Conclusions:** The cross-reactivity and sequence homology between α-synuclein and frequently consumed foods, reinforces the autoimmune aspect of Parkinson’s disease. It is hypothesized that luminal food peptides that share cross-reactive epitopes with human α-synuclein and have molecular similarity with brain antigens are involved in the synucleinopathy. The findings deserve further confirmation by extensive research.

## 1. Introduction

Environmental factors play a major role in human chronic diseases [1]. In addition to infections, chemicals and stress, nutritional behavior is emerging as an important factor, affecting the microbiome\dysbiome balance and their metabolome [2]. Food antigens are involved not only in allergy, but also contribute to autoimmune and neurodegenerative diseases [3,4]. Following ingestion, nutrients are digested into tolerated molecules that are absorbed for the benefit of human health and functions. Facing powerful physical barriers and immune mechanisms, immunogenic food antigens are denied entry, thus avoiding immune stimulation and self-reactions. If a non-naïve antigen circumvents those checkpoints, the mucosal innate and reactive immune systems respond, aiming to neutralize the invader. Indirectly, nutrients impact the human microbiome, thus prokaryotic constituents or their mobilome could penetrate the defense mechanisms and impact human health [5,6] If everything works perfectly, the resulting anti-food antibodies are directed to neutralize the foreign protein by forming a complex that will be eliminated by the cellular immune cells.

However, this complex formation between antibodies and food proteins can result in the activation of a complement cascade and binding to C1q. These anti-nutrient antibodies can cross-react with human self-molecules. This autoimmune cascade can also be evoked when sequence homology or shared immunogenic epitopes exist between those food-originated foreign proteins and the host’s self-determinants [7]. This phenomenon is called molecular mimicry or cross-reactivity, and is often described in various autoimmune conditions [7]. More and more such diseases are associated with various nutritional compounds [8]. Rheumatoid arthritis has been connected to wheat, fish, pork, milk and dairy products, eggs, lectins and agglutinins [7,9,10,11,12]. Multiple sclerosis incidence has been strongly correlated with the consumption of cow’s milk [12,13] and other food products [8]. Celiac disease, dermatitis herpetiformis and celiac ataxia are induced by gluten-containing food products [1,2,3]. Many other autoimmune diseases might benefit from a gluten-free diet [5,14,15,16] or other restrictive dietary regimens [17]. Even an autoimmune neurological disease like polyradiculoneuropathy has been induced by porcine brains, although in this case the exposure of the affected abattoir workers was through aerosolized antigens and not per oral intake [18]. Based on the above studies, it can be concluded that nutrients are associated with autoimmune diseases. Many of the studies were performed on animal models, thus causality and mechanistic pathways are far from being elucidated.

No less interesting is the relationship between neuroinflammatory, neurodegenerative or neuropsychiatric conditions and food consumption. In this regard, bovine milk and dairy products [12], gluten [1,4,19] and red meat [20] have been suggested as high-risk nutrients. Interestingly, milk and dairy products are also among the list of foods said to exacerbate Parkinson’s disease (PD) [21], while some nutritional diets, like the Mediterranean and quasi-vegan diets, might be protective or preventive against it [22,23].

A very logical suggestion was recently suggested by Riccio and Rossano [24]. They concluded that “what determines the organ specificity of the autoimmune-inflammatory process may depend on food antigens resembling proteins of the organ being attacked. This applies to the brain and neuroinflammatory diseases, as to other organs and other diseases.” For example, the late-embryo-abundant group III protein family that is found in plants and seeds such as soybean, wheat, tomato, peanuts and in crustaceans as well, share the epitopes of 11 amino acid residues with α-synuclein (aSN) [25,26,27,28,29,30]. Furthermore, vertebrates, especially fish, fowl and mammals, are the main source of aSN, which after their consumption, can potentially pass the enteric barrier and reach the human brain. In alignment studies, high degrees of sequence homology were found between the aSN of fish, fowl, pig, sheep and cow and the aSN of humans [31,32,33,34,35]. Interestingly, this human-like aSN has been found in the brain and many other animal body tissues and organs, including muscle, bone marrow and ovaries, and in cells such as erythrocytes, platelets, myocytes and neurons [34,36,37,38]. Based on this molecular mimicry between human aSN and aSN from food sources such as mammalian meat, chicken, fish, grains and some plants, we undertook the present study. To our knowledge, there are no current published studies on cross-reactivity and sequence homology between food antigens and aSN. Based on the current enteric origin theory of PD development [39,40,41], the association of high-risk food products with PD [42], and the numerous luminal sources of aSN [43], we hypothesized that frequently ingested foods, if improperly digested in the presence of broken barriers, may enter into the blood. The subsequent production of antibodies against them may contribute to α-synucleinopathy in patients with PD and other neurodegenerative disorders. More so, if cross-reactivity between them coexist with sequence similarity, the two pathways can join together in the process of the autoimmunogenesis in PD. In individuals with elevated levels of aSN antibody in the circulation, these antibodies may react with foods containing aSN, forming immune complexes that manage to cross the blood-brain barriers (BBB) into the brain, where they may induce the formation of Lewy bodies, the hallmarks of PD. All that being said, food is only one of many environmental factors, biological, chemical and pathogenic, that are suspected of being involved somehow in PD, and this mimicry and cross-reactivity between food and anti-aSN antibodies is a new challenging aspect of the disease that deserves further study.

## 2. Materials and Methods

### 2.1. Antibodies and Antigens

Monoclonal mouse IgG1 antibody made against recombinant aSN was purchased from R&D Systems, Minneapolis, MN, USA. Monoclonal rabbit IgG antibody made against aSN epitope 118–123, was purchased from Abcam, Cambridge, MA, USA.

### 2.2. Preparation of Food Antigens

For the preparation of the food antigens, food products in either raw or cooked form were purchased from the supermarket. Food proteins undergo structural epitope transformation when the food is cooked, and dietary preparation was conducted to reflect dietary proteins in either raw or cooked form as represented in typical human diets. A total of 180 different food extracts, representatives of various meats, seafoods, grains, fruits, vegetables, seeds, nuts, beans, spices, gums and more were prepared, in a process similar to the one used in our earlier study [44]. The different foods were ground at 4 °C in either 70% ethanol, or coco buffer containing 0.55 M of NaHCO3, 1 % NaCl pH 8.5. Each food item was kept on the stirrer for 4 h at 25 °C. After each food was stirred, the food processor was decontaminated. The mixture was centrifuged at 2000 g for 15 min, and then the top layer, which contained oil bodies, was discarded. To ensure that all small molecules were removed, the liquid phase from each solvent was dialyzed against a buffer of 0.01 M phosphate buffered saline (PBS) using dialysis bags with a cutoff of 6000 Da for 72 h, with the buffer changed every 24 h. Protein concentration was then measured using a kit provided by Biorad (Hercules, CA, USA). The list of the presently used 180 food’s Current Procedural Terminology (CPT) codes, developed by the American Medical Association, is presented in Supplementary Materials 1.

### 2.3. Enzyme Linked Immunosorbent Assay (ELISA) for Demonstrating the Reaction of Various Antibodies With Food Antigens

Food antigens at a concentration of one mg/mL were prepared. For coating the ELISA plate, the optimal concentration of each food antigen was determined by examining the concentration of antigens that gave the most reproducible results in quadruplicate. Accordingly, the stock solution was diluted from 1:50–1:200 in 0.1 M carbonate-bicarbonate buffer (pH 9.5). One hundred microliters were added to each well of the polystyrene flat bottom ELISA plate. The plates were then incubated overnight at 4 °C, and then washed 5 times with 200 microliters PBS containing 0.05 % Tween 20 at a pH of 7.4. 200 microliters of 2.5 % bovine serum albumin (BSA) were then added to prevent the nonspecific binding of immunoglobulins, after which the plates were incubated at 4 °C overnight. Plates were subsequently washed as described above, and then unimmunized mouse serum, rabbit serum, and mouse or rabbit monoclonal antibodies made against aSN at optimal dilutions of 1:100–1:300 were added to different groups of wells and were incubated at room temperature for 1 h. They were then washed 5 times with PBS-Tween buffer. We then added one hundred microliters of monoclonal antibody made against aSN, followed by incubation, washing, and the addition of secondary antibody or alkaline phosphatase labeled anti-rabbit or anti-mouse IgG to different wells, after which the plates were incubated again. 100 microliters of para-nitrophenylphosphate (PNPP) in 0.1 mL diethanolamine buffer 1 mg/mL containing 1 mM MgCl2 and sodium azide at a pH of 9.8 were added to start the enzymatic reaction, which was stopped 45 min later with 50 microliters of 1 N NaOH, so that the samples were ready for quantitative analysis by an ELISA reader. The microtiter reader recorded the optic density (OD) at 405 nm to provide quantitative antibody reactivity levels in comparison with control wells coated either with human serum albumin (HSA) as negative control or aSN as positive control. Each analysis was performed in quadruplicate.

### 2.4. Determination of Specificity of Antigen-Antibody Reaction

To demonstrate the specific binding of aSN antibodies not only to synuclein, but to different food antigens, serial dilutions and inhibition studies in the presence of different food antigens in the liquid phase of the antigen-antibody reaction was performed. Different sets of different ELISA wells were coated with the food antigens. In each set four or five foods were chosen as being representative of the antigens that showed from moderate to strong reactions with aSN antibodies. For the plates using mouse monoclonal antibody, these foods were honey, shrimp, yeast, tofu and quinoa. For the plates using rabbit monoclonal antibody, the foods were wheat germ agglutinin (WGA), lentil lectin, latex hevein, thyme and potato. The aSN antibodies were added serially in dilutions ranging from 1:100 to 1:12,800 to the appropriate set of microtiter strips. After incubation, washing, the addition of a second antibody, and the completion of other ELISA steps, the ODs were recorded at 405 nm.

The inhibition study was done by the addition of various concentrations of a specific antigen to an antibody in liquid phase first, followed by the addition of the antigen-antibody mixture to ELISA microwells coated with the same antigen. For example, to prove that the binding of aSN antibody to yeast, quinoa, tofu, shrimp and honey is specific, the following steps were taken:

1. Eight different ELISA plate wells were coated with optimal concentrations of yeast or another antigen used in this inhibition study.

2. 100 microliters of monoclonal anti-aSN were then added to each of 8 different test tubes.

3. The first tube, tube #1, containing anti-aSN antibody was to serve as the baseline reaction of antibody to each tested antigen, so no antigen (in this case, yeast) was added to this tube. To the other seven tubes containing anti-aSN antibody, we added increasing concentrations of yeast protein. Tube #2 received 2 micrograms, tube #3 received 4 micrograms, and tubes #4–8 received 8, 16, 32, 64, and 128 micrograms of yeast antigen respectively.

4. The eight tubes now contained 100 microliters of aSN-antibody and from 0 to 128 micrograms of yeast protein. The tubes were individually mixed, and then the contents of each tube were added to the appropriate ELISA wells coated with yeast protein described in Step #1.

After incubation, washing, the addition of anti-mouse or anti-rabbit IgG labeled with enzyme, and the completion of additional ELISA steps, the ODs were recorded. The inhibition of anti-aSN antibody binding to specific food antigen-coated ELISA wells by the same antigen in liquid phase was demonstrated graphically to be in proportion to the increased amount of protein used for inhibition. All of these same steps were also used for quinoa, tofu, shrimp and honey, which were the other food antigens used in the inhibition study using mouse monoclonal antibody.

The inhibition of anti-aSN antibody made against the short epitope AA 118–123 of aSN was done in a similar manner using food proteins that had moderate to strong reactions with aSN antibody.

### 2.5. Amino Acid Sequence Similarity Between Alpha-Synuclein and Food Proteins

We used the NIH/US National Library of Medicine’s Basic Local Alignment Search Tool (BLAST) sequence matching program to study degrees of possible mimicry between the amino acid sequences of aSN and food proteins such as soy bean, yeast, pea lectin, lentil lectin, bean agglutinin (concanavalin-A), wheat germ agglutinin, peanut protein, pineapple bromelain and shrimp. These are foods that had moderate to strong reactions with the aSN antibody. We also ran a sequence match search with proteins for coconut, a food that did not react with this antibody.

### 2.6. Statistical Analysis

Statistical analysis was performed using STATA 14.2 software. Independent *t*-tests were performed to evaluate mean differences of optical densities between controls and antigens. A Bonferroni adjustment was conducted to account for type 1 errors with multiple comparisons and alpha was set to <0.001.

## 3. Results

Based on earlier studies about the cross reactivity of food components and human tissue antigens [7,45,46,47,48,49], the present study explored the reactivity of aSN antibody with 180 different commonly used food products.

### 3.1. Cross Reactivity Between aSN and Different Food Antigens.

Anti-aSN antibodies had significant reactivity with 86/180 foods, much higher in comparison to the ODs of reagents, controls and the mean ODs of the other 94 foods +3SD (0.50).

**Figure 1:**
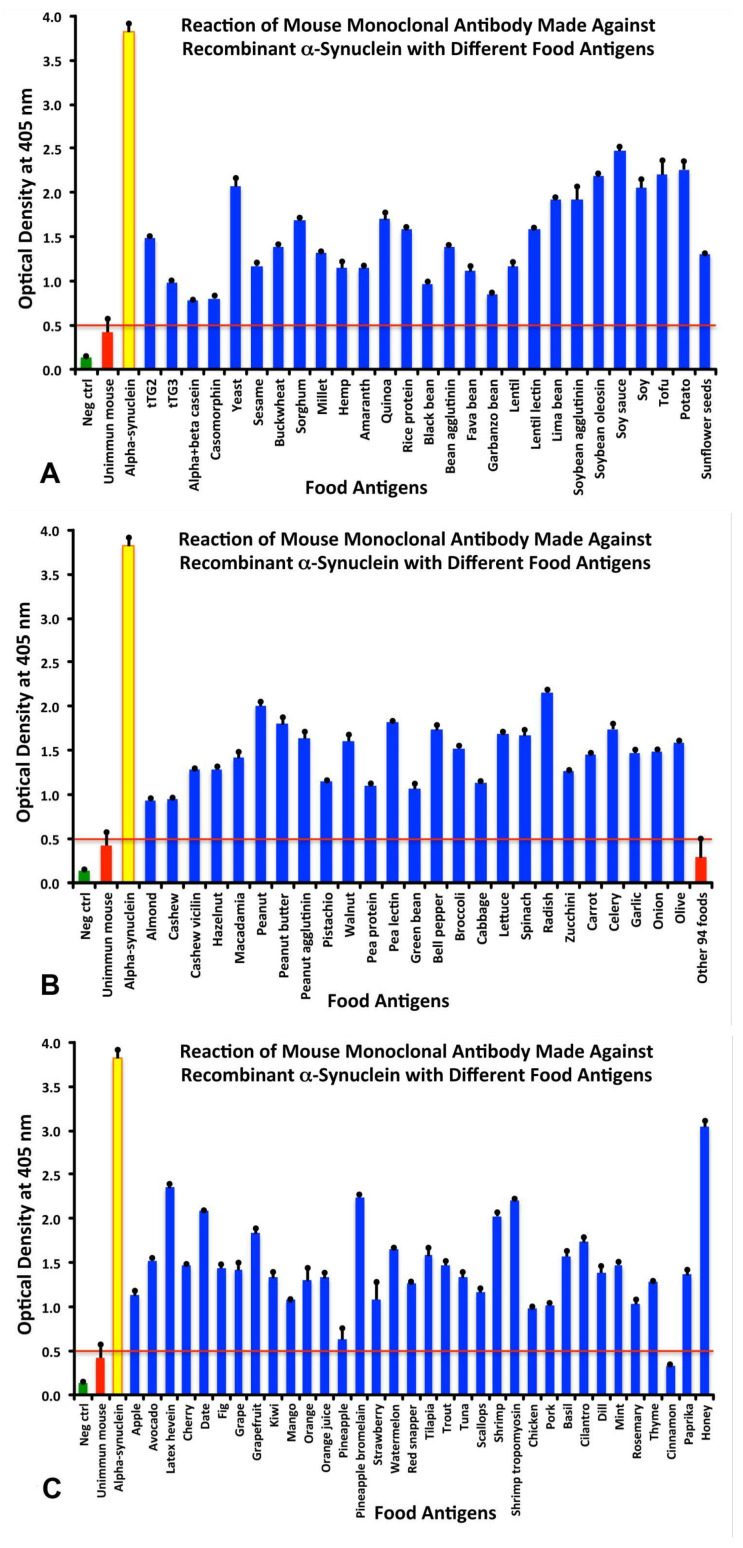
(**A**–**C**) Reaction of mouse monoclonal antibody made against recombinant aSN with different food antigens. tTG—tissue transglutaminase.

The resulting means +SD of each antigen’s quadruplicate ODs were calculated and are presented in Figure 1A–C. In order to semi-quantify the levels of aSN antibody’s reactivity with the studied food antigens, the following key for the ODs were used: 0–0.50 = insignificant or negative; 0.51–1.0 = low; 1.10–1.80 = moderate; 1.81–2.5 = high; and >2.5 = very high. The highest OD that can be obtained with the laboratory ELISA reader is 4.0.

Comparing the reaction of aSN antibody with alpha synuclein (3.82), which was close to the maximum OD of 4.0, the reaction of anti-aSN antibody was strongest with honey (OD 3.0—very high), followed by soy sauce (OD 2.5—high), potato (OD 2.3—high), tofu, radish and soybean oleosin (OD 2.2—high), yeast and soy (OD 2.1—high). Overall, not counting the highest reaction of aSN antibody with honey, 17 foods were graded as high (OD 1.81–2.5), 56 were graded as moderate, 12 were graded as low, and 94 foods had individual ODs that were equal to or less than the mean OD of all these 94 foods plus 3SD, or 0.50 or less, representing the insignificant or negative results (Figure 1A–C). Interestingly, the mean OD plus 3SD that resulted from the reaction between unimmunized mouse serum with all 180 food antigens was also very close to the above cutoff of 0.50 (Figure 1A–C). The difference between the ODs of the aSN-immune-reactive foods versus the non-immune reactive foods was very significant (*p* < 0.0001).

### 3.2. Cross Reactivity Between the Epitope 118–123 of aSN with Different Food Antigens

The aSN epitope 118–123 (VDPDNE) is localized at the polar, negatively charged and acidic C-terminal tail of aSN, representing a cleavage site, playing an important role in fibrillar synucleinopathy [50,51,52,53].

In order to semi-quantify the levels of the reactivity of this epitope’s antibody with the studied food antigens, the following key for the ODs were used: 0–0.51 = insignificant or negative; 0.52–1.0 = low; 1.10–1.80 = moderate; 1.81–2.5 = high; and >2.5 = very high. Whereas the complete sequence aSN protein antibody reacted significantly with 86 food antigens, the monoclonal antibody made against this specific epitope resulted in reactivity with a total of 32 food antigens, ranging from low to high reactivity.

**Figure 2:**
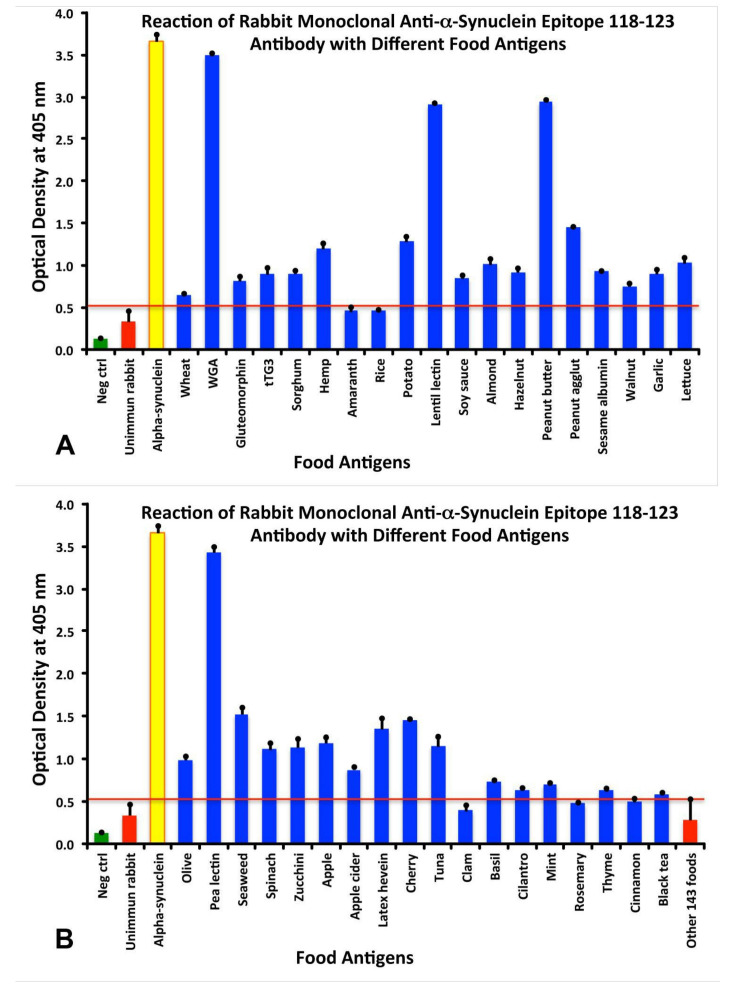
(**A**,**B**) Reaction of rabbit monoclonal antibody made against anti-epitope 118–123 with different food antigens. WGA—wheat germ agglutinin, tTG3—tissue transglutaminase 3.

The reaction of anti-aSN antibody was strongest with WGA (OD 3.5—very high), followed by pea lectin (OD 3.4—very high), peanut butter and lentil lectin (OD 2.9—very high). There were no food antigens in the high zone range, 10 food products in the moderate range, and 19 in the low range. All the rest of the tested food antigens were below the cut-off level of 0.52 and were considered negative (Figure 2A,B). The difference between the ODs of the aSN-immune-reactive foods versus the mean OD plus 3SD of the 148 non-immune reactive foods was very significant (*p* < 0.0001).

### 3.3. Analytical Specificity of Anti-aSN Antibody Binding to Selected Food Antigens Performed by Serial Dilutions

**Figure 3:**
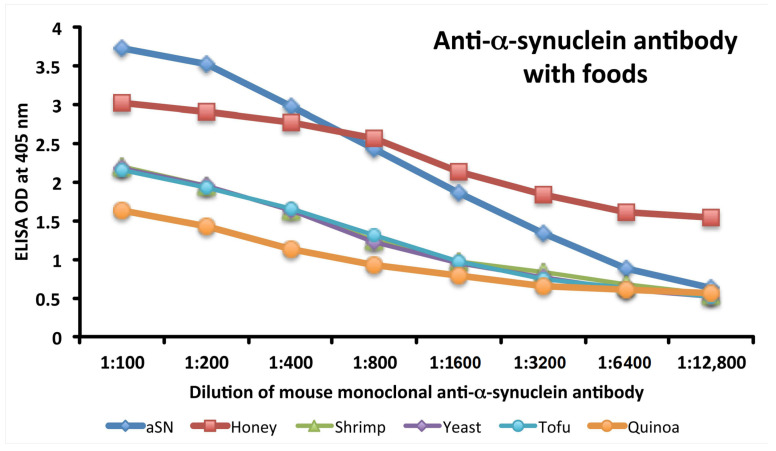
Analytical specificity by serial dilution study for mouse monoclonal anti-aSN. Shown are the reactions of various dilutions of mouse monoclonal anti-aSN antibody with alpha-synuclein (blue diamond), honey (red square), shrimp (green triangle), yeast (purple diamond), tofu (light blue circle) and quinoa (orange circle).

**Figure 4:**
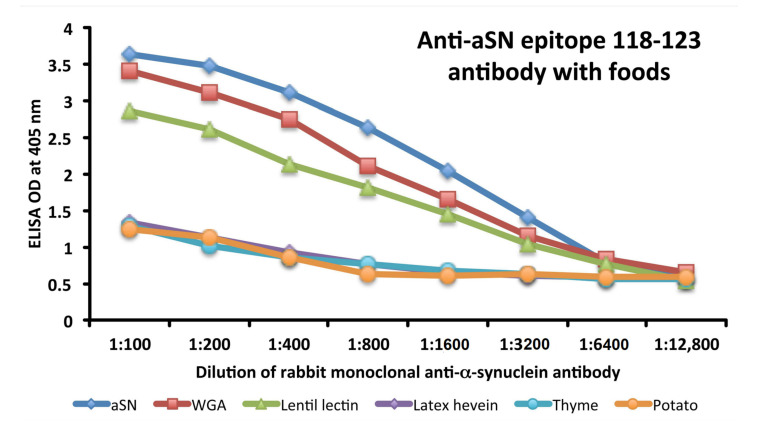
Analytical specificity by serial dilution study for rabbit monoclonal anti-aSN epitope 118-123. Shown are the reactions of various dilutions of rabbit monoclonal anti-aSN antibody with alpha-synuclein (blue diamond), WGA (red square), lentil lectin (green triangle), latex hevein (purple diamond), thyme (light blue circle) and potato (orange circle). aSN—alpha-synuclein, WGA –wheat germ agglutinin.

Specificity of anti-aSN antibody binding to various food antigens is demonstrated by serial dilutions for the complete aSN sequence in Figure 3 and for the short aSN epitope 118–123 in Figure 4. The addition of anti-aSN antibody, diluted from 1:100–1:12,800, to a fixed concentration of six different antigens is presented (aSN, yeast, quinoa, tofu, shrimp and honey for the complete aSN sequence, and aSN, wheat germ agglutinin, lentil lectin, latex hevein, thyme and potato for the aSN 118–123 sequence). Plates were incubated and followed by other required ELISA steps. After the addition of substrate and color measurement and calculation of indices, a significant decrease in antibody indices, in proportion to the antibody dilution, was observed. Logically, the highest decline of aSN antibodies was observed against the aSN itself, as a control. The other declines followed precisely their OD values, as shown in Figure 1A–C for the full aSN sequence, and Figure 2A,B for the aSN 118–123 sequence. More specifically, the very high cross reactivity of the aSN full sequence with honey (Figure 1C) had the steepest decline (Figure 3). Yeast, tofu and shrimp with their high OD levels (Figure 1A,C) had a middle decline (Figure 3), while quinoa, with its moderate cross reactivity to aSN (Figure 1A) assumed the lowest decline during the dilution experiment (Figure 3). For the aSN 118–123 sequence, it’s very high cross-reactivity with wheat germ agglutinin (OD 3.5, Figure 2A) had the steepest decline (Figure 4). Lentil lectin with an OD of 2.9 (Figure 2A) assumed a moderate decline, while latex hevein (OD 1.4, Figure 2B), potato (OD 1.3, Figure 2A) and thyme (OD 0.6, Figure 2B) had the lowest declines during the dilution experiment (Figure 4).

### 3.4. Analytical Specificity of Mouse Monoclonal Anti-aSN Antibody Binding to Selected Food Antigens Performed by Serial Inhibitions

**Figure 5:**
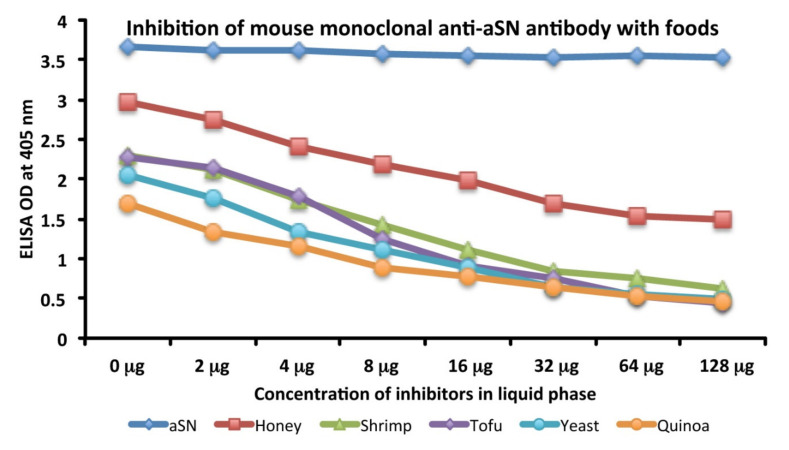
Analytical specificity by inhibition study for mouse monoclonal anti-aSN antibody binding to various food antigens. Graph shows the inhibition of mouse monoclonal aSN antibody with different concentrations of aSN (blue diamond), honey (red square), shrimp (green triangle), tofu (purple diamond), yeast (light blue circle) and quinoa (orange circle).

The binding of anti-aSN antibody to aSN as the control in the presence of HSA, or anti-aSN antibody binding to various cross-reactive food antigens was examined using different concentrations of cross-reactive food components, in a liquid phase. Figure 5 shows the inhibition of mouse monoclonal aSN antibody binding in the presence of different concentrations of HSA, or the binding of anti-aSN antibody to yeast, quinoa, tofu, shrimp and honey in the presence of 0–128 micrograms of yeast, quinoa, tofu, shrimp and honey. Compared to HSA, which caused no inhibition in this antibody-antigen reaction, the addition of higher concentrations of specific food antigens to the liquid phase, followed by the addition of aSN antibodies, resulted in a significant decline in the reaction of aSN antibodies to the cross-reactive-food-coated plates. This inhibition was observed even with a lower concentration of nutritional antigens, for example, 2–4 micrograms. Similar to the dilutional assay, the inhibition declines followed precisely their OD values, as shown in Figure 1A–C. More specifically, the very high cross reactivity to honey (Figure 1C) had the steepest decline (Figure 5). Shrimp, tofu and yeast with their high OD levels (Figure 1A,C) had a middle decline (Figure 5), while quinoa, with its more moderate cross reactivity to aSN (Figure 1A) assumed the lowest decline during the dilution experiment (Figure 5). A negative correlation was observed between the concentration of inhibitors in the liquid phase and the corresponding steepness of the antigenic food graphs (Figure 5).

### 3.5. Analytical Specificity of the Rabbit Monoclonal Anti-aSN Epitope 118–123 Antibody Binding to Selected Food Antigens Performed by Serial Inhibitions

**Figure 6:**
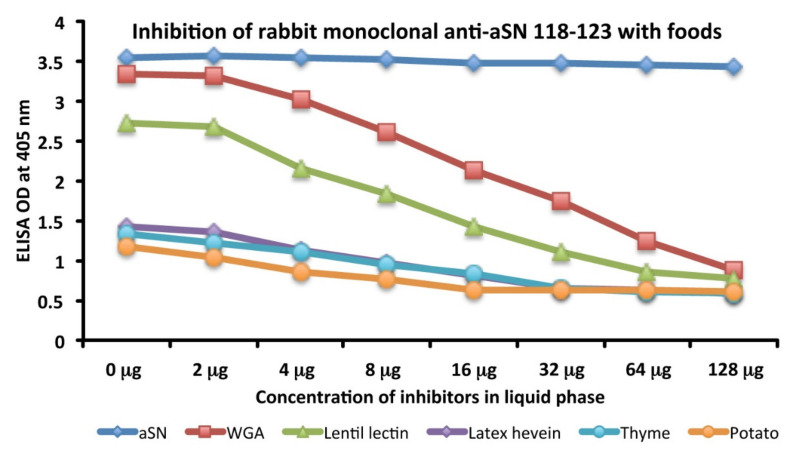
Analytical specificity by inhibition study for rabbit monoclonal anti-aSN epitope 118–123. Shown are the reactions of various dilutions of rabbit monoclonal anti-aSN antibody with aSN (blue diamond), WGA (red square), lentil lectin (green triangle), latex hevein (purple diamond), thyme (light blue circle) and potato (orange circle). aSN—Alpha-synuclein; WGA—wheat germ agglutinin.

The binding of rabbit monoclonal antibody binding to aSN and various cross-reactive food antigens was examined in the presence of different concentrations of HSA or the same food antigens in the liquid phase of the assay. Inhibition in the binding of rabbit monoclonal anti-aSN Epitope 118–123 antibody to 5 different food antigens and aSN are shown in Figure 6. The graph shows the inhibition of rabbit monoclonal anti-aSN antibody binding to aSN (blue diamond), WGA (red square), lentil lectin (green triangle), latex hevein (purple diamond), thyme (light blue circle) and potato (orange circle) with different concentrations (0–128 microgram) of the same antigen in liquid phase. Compared to HSA, which caused no inhibition in this antibody-antigen inhibition reaction, the addition of increasing concentrations of specific food antigens to the liquid phase, followed by the addition of aSN antibodies, resulted in a significant decline in the reaction of aSN antibodies to the cross-reactive-food-coated plates. This inhibition was observed even with a lower concentration of nutritional antigens, for example, 2–8 micrograms. Similar to the dilutional assay, the inhibition declines followed precisely their OD values, as shown in Figure 2A,B. More specifically, the very high cross reactivity to WGA (OD 3.5, Figure 2A) had the steepest decline (Figure 6). Lentil lectin, with an OD of 2.9, (Figure 2A) assumed a moderate decline, while latex hevein (OD 1.4, Figure 2B), potato (OD 1.3, Figure 2A) and thyme (OD 0.6, Figure 2B) had the lowest decline during the dilution experiment (Figure 6). A negative correlation was observed between the concentration of inhibitors in the liquid phase and the corresponding steepness of the antigenic food graphs (Figure 6).

### 3.6. Sequence Similarity Between Alpha-Synuclein and Food Antigens

Amino acid sequences for proteins from peanut, yeast, potato, shrimp, pineapple bromelain, bean agglutinin (concanavalin-A), pea lectin, lentil lectin, soybean agglutinin, wheat germ agglutinin, and latex hevein, which had moderate to very strong reactions with aSN antibody (Figure 1A–C), were downloaded from NCBI resources and were compared to the aSN 140 amino acid sequence using the NIH/US National Library of Medicine’s Basic Local Alignment Search Tool (BLAST) sequence matching program.

The food proteins with the greatest number of immunological peptides that matched with aSN were yeast (*Saccharomyces cerevisiae*), soybean agglutinin, latex hevein, wheat germ agglutinin, peanut, pea lectin, potato, bean agglutinin, and shrimp. Bromelain had two matches, while lentil lectin only had one, but all the other proteins had from four to more than twenty matches with aSN. Even when the cutoff was set for percentage of identity at 50%, the resulting number of matches for many of those proteins was so overwhelming that not all of the instances of similarity could practically be shown in a table (Table 1, Table 2, Table 3, Table 4, Table 5, Table 6, Table 7 and Table 8), as was the case with yeast and soybean agglutinin. Honey was the only food for which we were unable to find protein sequences from NCBI, and consequently we were unable to compare its sequence with the aSN one. Finally, in many instances, matches with food proteins occurred in more than one chain or section of the aSN sequence, as are marked with * in Table 1, Table 2, Table 3, Table 4, Table 5, Table 6, Table 7 and Table 8.

Coconut was one of the foods that did not react significantly with the aSN antibody. When we tried to match it with aSN anyway, we found only one promising match (KESGVINEKNIAE), but it only had a 46% match with the aSN sequence (KE-GVVAA---AE).

## 4. Discussion

Parkinson’s disease is characterized by the abnormal folding of aSN, a protein localized in the substantia nigra in the form of Lewy bodies. Regarding the source of misfolded aSN in the brain, the current hypothesis is that chemicals, infections or other unknown environmental factors penetrate the peripheral nervous system through the gastrointestinal system or through the olfactory bulb in the nasal cavity, subsequently moving on to the adjacent enteric nerve, from there finally reaching the brain, then culminating in widespread aSN pathology at the later stages [43,54,55,56]. The hypothesis is partially supported by the fact that many food sources, including plants and seeds such as soy bean, beans, peanuts, wheat and tomato, as well as crustaceans such as shrimp, contain different proteins that share high degrees of similarity with aSN [25,26,27,28,29,30]. Furthermore, aSN sequences are highly conserved in vertebrates, particularly in mammals. Thus, the consumption of animal food products originated from fish, fowl, and mammals such as cattle, pigs, sheep and goats, especially animal food products containing red blood cells, are the major source of aSN that reach the human gut [33,34,35,36,37].

In the present study, the hypothesis was that if aSN shares molecular similarity with so many foods, then monoclonal antibodies made against aSN should react with some of those food proteins. Looking at it from the other direction, if anti-aSN reacts with different food antigens, then amino acid sequence similarities should exist between those food proteins and aSN.

Following the above-mentioned hypothesis, it can be envisioned that if foods that cross-react with aSN are partially or undigested in the gut due to local eco events, the aSN in those food particles may become misfolded, and its handling by the enteric nerve may help in the transportation of the misfolded aSN into the brain. Based on the increased barriers permeability in PD, the leaky gut and the permeable BBB may also allow aSN aggregates to migrate cephalically [57].

Molecular mimicry between food antigens and self-tissue proteins is one of the possible mechanisms in the initiation of autoimmune diseases [7,8,45,46,47,48,49,58,59]. Intriguingly, based on this mechanism, several aspects of neuroinflammation and autoimmunity have been described in PD [60,61]. Confirmational molecular mimicry between curli, an *E. coli* protein fibril, and two other protein amyloids, silk and Sup35, produced by certain microbiota, was found to accelerate the aggregation of amyloid protein A and aSN in animal models of amyloidosis and PD [62,63]. Besides molecular mimicry involving microbes, viral infections like herpes simplex-1 and Epstein Barr virus have been detected in the blood samples of PD patients. Interestingly, antibodies targeting the virus’ proteins showed cross-reactivity to human aSN. The C-terminus of LMP1, a late membrane protein of the Epstein Barr virus, presents a strong amino acid sequence similarity to aSN C-tail [50,51,52,53]. However, except for the suggested molecular homology between microbiota or viral proteins and aSN [54,55] and the possibility that high intake of Western diet and red meat might increase the risk of PD [23,64,65], no study, to the best of our knowledge, is available on immunogenic cross-reactivity between numerous specific nutrients and aSN protein. Thus, it was logical to apply monoclonal antibodies made against recombinant aSN or its short epitope to a variety of food antigens commonly consumed by humans.

While antibody made against the short 118–123 epitope of aSN had low to strong reactions with 32 different food antigens, the monoclonal antibody that was prepared against the full-sequence recombinant aSN reacted with 86 out of the 180 tested food antigens. These reactive antigens were prepared from seeds, nuts, beans, fruits, vegetables, seafood, fish, chicken and mammalian meats (Figure 1A–C). The observation that the reaction of anti-aSN antibody was strongest with honey (OD 3.0—very high, Figure 1C) deserve some explanation. Honey is very high in sugars but other constituents are various organic acids, enzymes (invertase, insulase), traces of vitamins and proteins and pollen grains originated from flowers are also found in honey. The anti-aSN-honey cross reactive antibodies, most probably, are directed to the protein part, however, in this assay, sugar moieties might exert an effect. This extensive immunoreactivity between aSN antibodies and so many food antigens may indicate that cross-reactivity through mimicry exists between food-sourced aSN and human aSN.

The reactivity of aSN antibodies with many food antigens was confirmed by the high degree of peptide sequence homologies between aSN and yeast, soybean agglutinin, wheat germ agglutinin, latex hevein, potato, peanut, bean agglutinin (concanavalin-A), pea lectin, shrimp, pineapple bromelain, and lentil lectin (Table 1, Table 2, Table 3, Table 4, Table 5, Table 6, Table 7 and Table 8). Using the BLAST matching program, a multiplicity of protein sequences in all those food proteins that showed varying degrees of similarity with aSN, were detected. The cutoff at an ID percentage of 50% was applied, so that only listed matches of 50% or higher were listed. In many instances, repeating sequences were observed in different subunits of the food proteins and the aSN sequences. The highest homology was found between aSN and yeast, followed by soybean agglutinin, WGA, latex hevein, potato, peanut, concanavalin-A, pea lectin, shrimp, bromelain, and lentil lectin. As a contrary proof, coconut protein, which did not react at all with aSN, predictably had no significant homology with aSN. Honey, on the other hand, reacted very strongly with aSN antibody, but we were unable to find a usable protein sequence for honey in the matching program index.

The plethora of these matches between aSN sequences and food antigens may explain why monoclonal antibodies made against aSN proteins reacted with so many food antigens out of the 180 in the present study. It should be noted that the study was limited to the identification of general cross-reactive antibody responses, while the BLAST search was just limited to 12 food antigens. The results may indicate that the aSN antibodies reacted against shared conformational epitopes in the food proteins. The current study design did not specifically include analyses that would capture conformational or non-linear epitopes, but screened any of the food sequences that matched with the aSN sequences, especially the highly recurring ones, hence possibly be conformational epitopes. Conformational epitopes could be major targets of autoantibody production in autoimmune diseases [66,67], resulting in aSN aggregation and associated pathology. Further investigation to identify the specific cross-reactive epitopes will require specific peptide fragmentation inhibition studies, epitope mapping, as well as computational modeling, which were not within the scope of this study.

The present results open a new horizon for food specific antigens that might drive PD. It is a new insight that reflects a connection between the disease and commonly consumed foods that enter the gut lumen, where they are digested, absorbed, sensed and sampled by the pluripotent epithelial and sub-epithelial innate immune cells [6,39]. The reactive mucosal immune B lymphocyte is activated, directly or indirectly, and responds by producing IgG isotype antibodies specifically directed against the nutritional antigens. Those food-specific antibodies, by cross-reacting with so many aSN sequences (Table 1, Table 2, Table 3, Table 4, Table 5, Table 6, Table 7 and Table 8), may help not only in the aggregation of aSN but also its delivery to the brain through the enteric nerve, and the broken gut and BBBs.

In support of the dual-hit hypothesis of PD by Braak et al. [54,55,56], Chandra et al. [68] suggested that under the influence of various environmental factors, aSN becomes misfolded in the gut endocrine cells, where, through its communication with the adjacent enteric neurons, the aSN starts its propagation up to the brain [69]. Interestingly, the enteric glial cells influence and are influenced by the enteric epithelial cell, gut microbiome, nutrient content and mucosal immunity. Taken together, those influences might affect tight junction functional integrity and facilitate aSN gut’s permeation [69,70,71,72,73]. Moreover, the gut is very rich in enzymes, both originated from dietary sources, or secreted as the result of gut dysbiosis, which contributes to post-translational modification of proteins (PTMP), including aSN [6,74,75,76,77].

Like many other proteins in the gut, aSN may undergo PTMP by acetylation, phosphorylation, oxidation, ubiquitination, glycation, nitration, deamidation, transamidation and sumoylation. This enzymatic PTMP reaction can result in the alteration of protein structures, affecting their functional and aggregatory capacity [76,77]. Furthermore, luminal aSN aggregation might be enhanced by the interaction of food-sourced aSN and late embryo-abundant (LEA) in the gut with toxic chemicals in the foods, as well as by compounds originated from disturbed gut microbiota, such as bacterial toxins, amyloids, and antibodies in the lumen [43,78,79,80,81,82]. The aggregation of luminal aSN and its entry into the enteric nervous system has been shown to be the next important step in delivering those detrimental molecules into the brain [83].

Simultaneously, aggregated aSN, microbial generated toxins, antigens, amyloids, antibodies, inflammatory cytokines, and undigested food antigens can affect the functional integrity of the tight junctions [3,6,39,43]. The failure of the tight junction integrity results in the entry of those macromolecules into the submucosa, the regional lymph nodes, and into the circulation. An immune response against them results in the production of cytokines and antibodies. The reaction of these antibodies with additional antigens results not only in antibody-antigen binding, but also in the activation of a complement cascade. Together, those two may affect the integrity of the BBB, the regulatory gatekeepers of the brain. Indeed, increased intestinal and BBB permeability has been described in PD and other neuroinflammatory and neurodegenerative disorders [6,84,85,86,87,88,89,90,91,92,93].

Although the presence of some of these factors (antigens, antibodies, immune complexes, aggregated asN, inflammatory cytokines) in the blood may not be directly pathogenic for the brain, it is possible that in the context of BBB permeability, these factors may contribute to the severity of diseases in the brain [87]. Thus, the gut is an ignition source for aggregated aSN and its delivery to the brain, not only by the enteric nervous system, but also by permeable gut and BBB, which altogether, contribute to PD synucleinopathy [43,78,84,88,89].

It is not known if the cross-reactive food specific antibodies against aSN epitopes will induce gain or loss of aSN functions. Most recently, strategies to decrease synucleinopathy by tackling the aSN molecule have been described, including targeting the spread, production, aggregation, and degradation of aSN [94]. One wonders what effects the binding of cross-reactive nutrient-specific antibodies to aSN might have on those functions. For example, specific antibodies could potentially neutralize aSN monomers and/or aggregates extracellularly or even intracellularly, as suggested in the case of intra/nanobodies [95]. Targeted aSN immunotherapy could become a shifting paradigm and disease-modifying therapeutic protocol.

**Figure 7:**
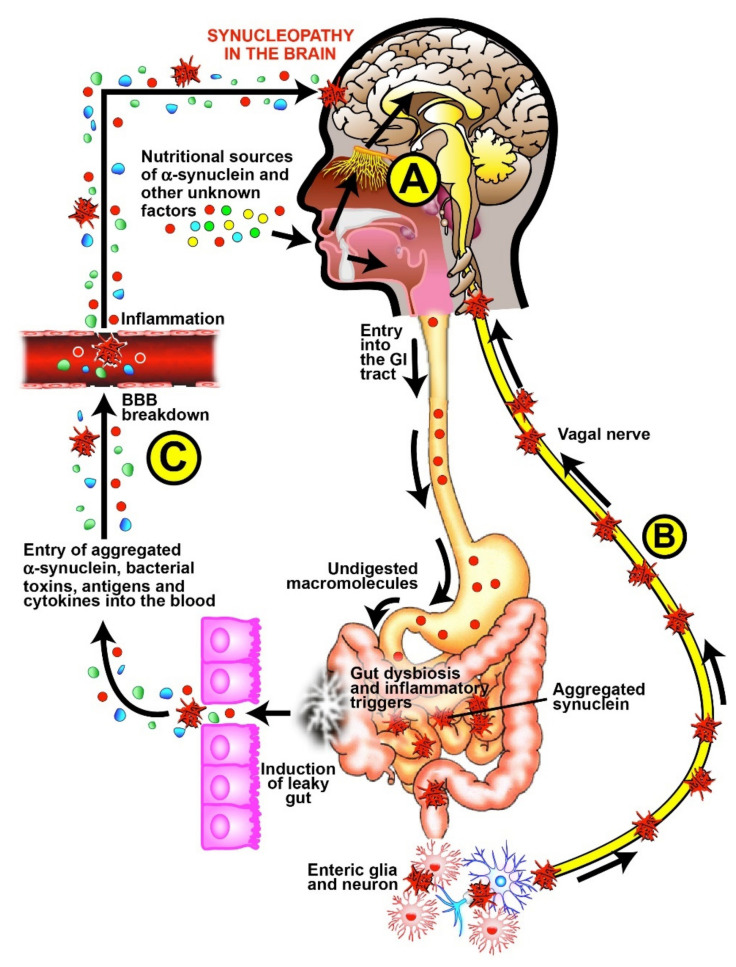
The triple-hit hypothesis of alpha-synucleopathy in the gut-brain axes of patients with Parkinson’s disease. Nutritional sources of aSN or cross-reactive nutritional peptides enter in the oral cavity. After chewing, some of those transformed substances may penetrate through the olfactory bud in the nasal cavity into the brain (**A**). The nutritional factors proceed from the oral cavity into the stomach and intestine. If improperly digested or modified, those nutritional substances may induce gut dysbiosis, enhance the production of inflammatory triggers and amyloidogenesis resulting in the aggregation of aSN. The aggregated aSN activates enterocytes/enteroendocrine/subepithelial dendritic cells, in close contact with the enteric glia cells and through the neurons, uptake the aSN aggregates and deliver them to the brain via the vagal nerve (**B**). The inflammatory mediators trigger leaky gut and enhance the entry of aggregated aSN and other macromolecules into the circulation, igniting further inflammation in the blood. Those molecules together induce breakdowns in the BBB, followed by the entry of the aggregated aSN and other molecules into the brain (**C**). Thus, the entry of aggregated aSN via the olfactory bulb, the enteric nerve, or broken gut and blood-brain barriers can result in synucleopathy and the formation of Lewy bodies in Parkinson’s disease.

Several consequences of aSN antibodies cross-reacting with a variety of food proteins or specific peptides can be postulated:

1. The presence of antibodies in the blood that cross-react with aSN may interfere with the accurate measurement of aSN antibodies used in support of PD diagnosis and progression assessment of the disease.
2. Compromised BBB, which is often observed in PD patients, may result in the entry of food cross-reactive antibodies into the central nervous system, where their binding to aSN can further contribute to Lewy body pathology.
3. The presence in the blood of food antigens that cross-react to aSN may interfere with treatment modalities in which anti-aSN antibodies are used to target misfolded proteins in PD.
4. High cross-reactivity of a food-specific antibody with aSN may affect some physiological functions of aSN, thus putting individuals at risk for synucleinopathy.
5. Glutamate neurotransmission and glutamate receptors play a pivotal role in brain physiology and are altered in PD [96,97]. On the other hand, the 33-mer peptide (p57–89) from the a2-gliadin subtype is considered the supramolecule that induces celiac disease [98]. Intriguingly, the component of the NMDA glutamate receptor ion channel—the human GRINA protein has a significant sequence homology with the 33-mer gliadin peptide [99]. The authors suggested that 33-mer gliadin molecule is a natural antagonist interfering with the normal interactions of GRINA, thus impacting the extraintestinal manifestations of celiac disease. This is a pathophysiological example of how molecular similarity between a luminal gluten-originated nutrient might affect PD behavior through the human glutamate receptor GRINA protein. In addition, celiac disease has been associated with “leaky gut” syndrome, an increase in the permeability of the intestinal mucosa [100]. It has also been shown that dietary imbalance impairs BBB properties, potentially favoring transmigration and leading to neuroinflammation [101]. Figure 7 summarizes schematically the triple-hit Hypothesis of alpha-synucleopathy in the gut-brain axes of patients with Parkinson’s disease.

It should be stressed that those possibilities need further investigation. There are some limitations for the present study. It is an in vitro study, but there would definitely be advantages in investigating in vivo, by feeding or injecting the aSN-cross-reactive food epitopes into animal models and observing if they develop PD-like symptoms or pathology. Ingested nutrients are subjected to enzymatic digestion in the stomach and small intestine. The post-digestion antigenicity of the presently studied food products were not explored. As it is, the clinical and pathophysiological impacts of the cross-reactive antibodies were not explored, and thus any conclusions are purely hypothetical, and it is not truly known whether aSN-cross-reactive food epitopes are pathogenic or protective. The strengths of the study are the plethora of food products, which span a wide range of common nutrients. The specificity of the antibodies’ level determination was double checked by dilution, inhibition and epitope similarity. Furthermore, the putative cross-reactivity between aSN and food proteins was supported by ELISA testing and BLAST matching with many lectins, agglutinins and different forms of the foods, such as soybean, soy sauce and tofu, peanut, peanut agglutinin and peanut butter.

Interestingly, in addition to the luminal aSN [34,36,37,38], another frequently ingested nutrient, namely, gluten, was recently suggested as a potential driver of neurodegeneration [100]. The anti-gluten antibodies cross-reactivity [7,46,47,59] and the numerous epitope sequence homologies with human central nervous system peptides [102] direct to the possible pathophysiological pathway of molecular mimicry, operating in neurodegenerative diseases. The food-gut-brain axis might represent an additional avenue, operating in neurodegenerative disease evolvement.

## 5. Conclusions

Multiple commonly consumed specific food products induce antibodies that cross-react with aSN and its 118–123 sequence. Our findings raise the possibility that antibody’s cross-reactivity supported by specific sequence’s molecular mimicries between nutrients and aSN is operative thus might represent an additional autoimmune feature to PD neurodegeneration. If substantiated in vitro or on PD animal models or in human subjects, this study’s findings might open a new avenue of research in PD pathophysiology. While the biological and pathophysiological cross-reactive and sequence homology effects are far from being unraveled, it is hoped that the current findings will encourage the scientific and clinical communities to explore the food cross-reactive and molecular mimicry phenomena in the PD enigma.

## Figures and Tables

**Table 1:** Potential cross-reactive epitopes between alpha-synuclein proteins and yeast proteins.

Yeast Protein	Yeast Sequence	Mapped Start To End	Alpha-Synuclein Sequence	ID (%)
Structure of the *S. cerevisiae* Srs2 C-terminal domain in complex with PCNA conjugated to SUMO	MDIFSQ-LSRAK	45–55	MDVFMKGLSKAK	58
Proteasome Activator Complex	KATNGVVIATE	19–29	KAKEGVVAAAE *	64
Structure of the ribosomal 80S-eEF2-sordarin complex	FMKKLRAAK---LAAPE	10–23	FMKGLSKAKEGVVAAAE *	53
Crystal Structure of the S. cerevisiae glucokinase, Glk1	MEKGLAPPKEG	43–53	M-KGLSKAKEG	64
Solution structure of the oxidized form of the Yap1 redox domain	MAKAKCSERGVVINAE	60–75	LSKAK--E-GVVAAAE	56
Cryo-EM Structure of the RIX1-REA1 Pre-60S Particle	KG-SKAK-GVNMAA	471–482	KGLSKAKEGV-VAA	71
Crystal structure of yeast elongation factor 2 in complex with sordarin	SKAGEIVLAA	813–822	SKAKEGVVAA *	70
Solution structure of the SBDbeta domain of yeast Ssa1	KGRLSKEDIEKMVAEAE	123–139	KG-LSKAK-EGVVAAAE	59
Crystal structure of the RNA recognition motif of yeast eIF3b residues 76–161	EATGKTK-GFLFV	48–59	EAAGKTKEGVLYV *	69
Structure of the ribosomal 80S-eEF2-sordarin complex from yeast	TKEAAAVAEGKSKQ	9–22	TKQGVAEAAGKTKE *	50
Crystal structure of a Prp8 C-terminal fragment	KQNDEEAAGASTVMKTK	276–292	KQGVAEAAG-----KTK *	53
Crystal Structures of Yeast Transketolase In Complex with Analogs of Thiamin Diphosphate	TPEGVAERAQKT	650–661	TKQGVAEAAGKT *	67
Crystal Structure of Yeast Aspartyl-Trna Synthetase Complexed with Trna Asp	KEGI-EMLRAAGK	336–347	KQGVAE---AAGK	54
A Gated Channel into The Proteasome Core Particle	TKEGVVLGV----EK	42–52	TKEGVVHGVATVAEK *	67
Structure of The Yeast Cytochrome Bc1 Complex Co-Crystallized with An Antibody Fv-Fragment	GSRYATKDGVAH	22–33	GSK—TKEGVVH *	58
Electron cryomicroscopy structure of *S. cerevisiae* FAS in the Apo state	SKTIKD-LVGGKSTV	169–182	SKT-KEGVVHGVATV *	53
Structure of the Ndi1 protein in complex with the competitive inhibitor, stigmatellin	VHLRTAVAKVEEK	271–283	VH---GVATVAEK *	54
Cryo-EM Structure of the Exocyst Complex	QVNSIGGVVVT	781–791	QVTNVGGAVVT	64
Arx1 pre-60S particle	NVGGALRVPG--AISEK	44–58	NVGGAV-VTGVTAVAQK	53
Crystallographic snapshots of eukaryotic dimethylallyltransferase acting on tRNA	IAGTTG-VGKSQL	7–18	IAAATGFVKKDQL *	62
Crystal structure of yeast aminopeptidase 1 (Ape1)	TAEGYGRIAVA	104–114	TVEGAGSIAAA *	64
Yeast RNA polymerase III initial transcribing complex	DNEDNE-GSEE-----EPE	579–591	DNEAYEMPSEEGYQDYEPE *	53

* This match occurred in more than one section of the target sequence. Not all of the instances of identification and similarity are shown due to the overwhelming number of occurrences.

**Table 2:** Potential cross-reactive epitopes between alpha-synuclein proteins and soybean agglutinin proteins.

Soybean Agglutinin Protein	Soybean Agglutinin Sequence	Mapped Start to End	Alpha-Synuclein Sequence	ID (%)
Uncharacterized protein LOC100790514 [Glycine max]	MNGLSK---GVVATA	178–189	MKGLSKAKEGVVAAA	67
Uncharacterized protein LOC100799919 [Glycine max]	GL-KTKEGVVLAVE	39–51	GLSKAKEGVVAAAE	71
Uncharacterized protein LOC100793479 isoform X1 [Glycine max]	FSTGLSKRTSKVAEKKEGTVAGA	79–101	FMKGLSK-----A--KEGVVAAA	52
Coleoptile phototropism protein 1 [Glycine max]	MDYFVKTLSGIKAK-GV	57–72	MDVFMKGLS--KAKEGV	65
Proteinaceous RNase P 2 [Glycine max]	VGEAEFDAGRVKEGVL	219–234	VAEA---AGKTKEGVL	63
Uncharacterized protein At4g13230 [Glycine max]	KTKES-AEHAKDNVVGKTKESAEYV	121–144	KTKQGVAEAA-----GKTKEGVLYV	56
Soyasapogenol B glucuronide galactosyltransferase-like [Glycine max]	KT-QG--EEEGWLEWLNKKKEGSVLYV	268–291	KTKQGVAEAAG------KTKEG-VLYV	52
Peptidyl-prolyl cis-trans isomerase FKBP62 [Glycine max]	EAAGKKKEEGNVLF	389–402	EAAGKTKE--GVLY	64
51 kDa seed maturation protein isoform X1 [Glycine max]	VGDAAQKTKE---YV	164–175	VAEAAGKTKEGVLYV	60
Binding partner of ACD11 1 [Glycine max]	KTKEKVLAQDNQGKTEEG	228–245	KTKQGV-AEAA-GKTKEG	56
ATP-dependent DNA helicase Q-like 3 isoform X1 [Glycine max]	SKIK--VV--VATVA	307–317	SKTKEGVVHGVATVA	67
Hypothetical protein GLYMA_15G213400 [Glycine max]	GVVQHGPGGTMATVAE	1008–1023	GVV-HG----VATVAE	63
Proteasome subunit alpha type-5 [Glycine max]	KTKEGVVLAV----EK	41–51	KTKEGVVHGVATVAEK	63
Subtilisin-like protease SBT1.2 [Glycine max]	VTNVGEANSSYVVT-VSA	692–708	VTNVGGA----VVTGVTA	61
AT-hook motif nuclear-localized protein 23 [Glycine max]	VTNVSLRQPASAGAVVT	112–128	VTNVG-------GAVVT	53
NAD-dependent malic enzyme 59 kDa isoform, mitochondrial [Glycine max]	VTAEVGAAVV--CAAVAEK	543–559	VT-NVGGAVVTGVTAVAQK	58
Subtilisin-like protease SBT5.3 [Glycine max]	VTNVGKARSIYKAVVVSPTGVNVTVV	679–704	VTNVG------GAVV---TG--VTAV	50
Pyruvate decarboxylase 2 [Glycine max]	VE---AIQTATG-VKKD	555–567	VEGAGSIAAATGFVKKD	59
2-oxoglutarate dehydrogenase, mitochondrial [Glycine max]	SAATATGFLKVHQKEQ	993–1008	SIAAATGFVK---KDQ	56
Pyruvate decarboxylase 2 [Glycine max]	VE---AIATATG-PKKDSL	560–574	VEGAGSIAAATGFVKKDQL	58
Autophagy-related protein 18f isoform X2 [Glycine max]	TVSGAA--AAATG--RKNAL	572–587	TVEGAGSIAAATGFVKKDQL	55

* This match occurred in more than one section of the target sequence. Not all of the instances of identification and similarity are shown due to the overwhelming number of occurrences.

**Table 3:** Potential cross-reactive epitopes between alpha-synuclein proteins and latex hevein proteins.

Latex Hevein Protein	Latex Hevein Sequence	Mapped Start to End	Alpha-Synuclein Sequence	ID (%)
Hypothetical protein GH714_008467	LSKAKGGVVAEEE	28–40	LSKAKEGVVAAAE	77
Hypothetical protein GH714_000288	KGVLSRKAKEAAVAAAQ	176–192	KG-LS-KAKEGVVAAAE *	71
Hypothetical protein GH714_000288	KENVVEA	587–593	KEGVVAA *	71
Uncharacterized protein LOC110639732	LSLKAHDGGVVA	257–268	LS-KAKEG-VVA	67
Lysine-specific histone demethylase 1 homolog 1-like	MKG-----DGVVAAAD	288–298	MKGLSKAKEGVVAAAE *	56
Proteasome subunit alpha type-5	GL-KTKEGVVLAVE	39–51	GLSKAKEGVVAAAE	71
Hypothetical protein GH714_036586	LDVFMKGI--AAEG	223–234	MDVFMKGLSKAKEG	64
Hypothetical protein GH714_025383	DVFMKGI--AAEG	282–292	DVFMKGLSKAKEG *	69
Hypothetical protein GH714_028032	DVFTKSLSQAK	237–247	DVFMKGLSKAK *	73
ABC transporter B family member 2-like	KEGASEGEVVEAA	1096–1108	KEG-----VVAAA	54
E3 ubiquitin-protein ligase CIP8	YELPTDD--QDYE	288–298	YEMPSEEGYQDYE	54
The article Zinc finger protein BRUTUS-like At1g18910	EAYEMPYASE	583–592	EAYEMP—SE *	80
DEAD-box ATP-dependent RNA helicase 56-like isoform X1	DNDAYE---EE-LLDYEEE	6–20	DNEAYEMPSEEGYQDYEPE *	58
Hypothetical protein GH714_022075 [Hevea brasiliensis]	EAYESSSEESFKD	180–192	EAYEMPSEEGYQD	62
Hypothetical protein GH714_005670	DNERFDF-NEEPYQQYE	231–246	DNEAYEMPSEEGYQDYE	53
Splicing factor U2af large subunit B-like isoform X4	YE---EEGYQGNGEDFE	4–17	YEMPSEEGYQ----DYE	53

* This match occurred in more than one section of the target sequence. Not all of the instances of identification and similarity are shown due to the overwhelming number of occurrences. Only identifications with the sequences from 1–20 and 121–140 of the complete Latex hevein sequence are shown.

**Table 4:** Potential cross-reactive epitopes between alpha-synuclein proteins and wheat germ agglutinin.

Wheat Germ Agglutinin Protein	Wheat Germ Agglutinin Sequence	Mapped Start to End	Alpha-Synuclein Sequence	ID (%)
Chain A, Glutaredoxin	LAKAKE-IVASA	53–63	LSKAKEGVVAAA	67
Chain Y, 60s Ribosomal Protein L24	KAKGKFTAEDV-AAA	129–142	KAK-----EGVVAAA	53
Chain I, 60s Ribosomal Protein L16	DVGMK-----KKGV	35–43	DVFMKGLSKAKEGV	50
Chain F, 60s Ribosomal Protein L30	KQKVAAEKIKAAENTK--VIY	29–47	KQGV-AE---AAGKTKEGVLY	52
Chain u, 60s Ribosomal Protein P1	GGAAAAEEKKE	80–90	GVAEAAGKTKE	55
Chain A, Xylanase Inhibitor Protein I	VHPKNVYYGVAPVAQK	231–246	VH------GVATVAEK	50
Chain H, 60s Ribosomal Protein L6	EGVTVQ-VAAKVVTV	14–27	EGV-VHGVA----TV	53
Chain M, 40s Ribosomal Protein S12e	VHLV-TVPSAKT	114–124	VHGVATV-AEKT	58
Chain X, Xylanase Inhibitor	GSKPVSKVNV--GV	109–120	GSK—TKEGVVHGV *	50
Chain A, Eukaryotic Translation Initiation Factor 4e-1	GAVV----SVRQK	114–122	GAVVTGVTAVAQK	54
Chain A, Ribulose bisphosphate carboxylase large chain	EEAGAAVAAESSTG	51–64	EGAGS-IAA--ATG	50
Chain J, 60s Ribosomal Protein L5	GIQEHIDLGMKYDP	107–120	GILE--D--MPVDP	50

* This match occurred in more than one section of the target sequence. Not all of the instances of identification and similarity are shown due to the overwhelming number of occurrences.

**Table 5:** Potential cross-reactive epitopes between alpha-synuclein proteins and pea lectin, concanavalin-A (bean agglutinin) and lentil lectin proteins.

**Pea Lectin Protein**	**Pea Lectin** **Sequence**	**Mapped Start to End**	**Alpha-Synuclein** **Sequence**	**ID (%)**
Chain A, Ferredoxin—nadp reductase, leaf isozyme, chloroplastic	VYMMGL-KGME	263–272	VFMKGLSKAKE*	55
Chain A, Putative aminoaldehyde dehydrogenase	KEDVDVAVAAA	44–54	KEGV---VAAA	64
Chain B, Photosystem II CP47 reaction center protein	EGVAAA	94–99	EGVVAA	83
Chain X, Photosystem II reaction center protein X	KASLKEKVVTGLTAAA	16–31	KA--KEGVV----AAA	56
Chain A, Dihydrolipoamide dehydrogenase	KAEEDGVA	327–334	KAKEGVVA	63
Chain A, Dihydrolipoamide dehydrogenase	FTSGLNLDKIGV	280–291	FMKGLSKAKEGV	50
Chain A, Protein (ferredoxin: nadp + reductase)	VYMCGL-KGME	263–272	VFMKGLSKAKE *	55
**Concanavalin-A Protein**	**Concanavalin-A Sequence**	**Mapped** **Start to End**	**Alpha-Synuclein** **Sequence**	**ID (%)**
Chain A, Alpha-mannosidase	KAYEGEV	518–524	KAKEGVV	71
Chain A, Alpha-mannosidase	VENVLDSVV	48–56	VTNVGGAVV	56
Chain A, Urease	GALSIA----FVSKAALDQ	758–772	GAGSIAAATGFVKK---DQ *	53
Chain A, Refined crystal structure of cavavalin from jack bean	EE---QEGVIVKMP	168–178	EEGAPQEGILEDMP *	50
Chain A, Urease	KEEEDA-SEGV	24–33	KNEEGAPQEGI	55
Chain A, Alpha-mannosidase	YE--SSEGDFSDYQ	609–620	YEMPSEEG-YQDYE *	50
**Lentil Lectin Protein**	**Lentil Lectin** **Sequence**	**Mapped Start to End**	**Alpha-Synuclein** **Sequence**	**ID (%)**
Solution structure of Lipid Transfer Protein	AGSITKLNTNNAAA	56–69	AGSI-------AAA *	50

* This match occurred in more than one section of the target sequence.

**Table 6:** Potential cross-reactive epitopes between alpha-synuclein proteins and potato proteins.

Potato Protein	Potato Sequence	Mapped Start to End	Alpha-Synuclein Sequence	ID (%)
The structure of a glutathione synthetase (StGSS1) from *Solanum tuberosum* in ADP and y-EC bound closed conformation	SSSNEGGVAA	463–472	SKAKEGVVAA	60
Crystal structure of S-adenosylmethionine decarboxylase	KGLRSLSKA	35–43	KGL---SKA	67
Crystal structure of potato Rx-CC domain in complex with RanGAP2-WPP domain	LSKE-E---AAKNAE	36–46	LSKAKEGVVAA--AE	53
Structure determination and refinement at 1.8 A resolution of Disproportionating Enzyme	EGAVSSVARIA	458–468	EGVVHGVATVA *	55
The structure of a glutathione synthetase (StGSS1) from *Solanum tuberosum* in ADP and y-EC bound closed conformation	GVDMVH--APVA	54–63	GV--VHGVATVA	58
Characterization of *Solanum tuberosum* Multicystatin and Significance of Core Domains	AATDGG--KK	60–67	AAT--GFVKK	60
>Crystal Structure of Potato Multicystatin	>AATDDAG--KK	>55–63	>AAT---GFVKK	>55

* This match occurred in more than one section of the target sequence.

**Table 7:** Potential cross-reactive epitopes between alpha-synuclein proteins and peanut proteins.

Peanut Protein	Peanut Protein Sequence	Mapped Start to End	Alpha-Synuclein Sequence	ID (%)
Chain A, PR 10 protein	GLFRAIEGYVLA	140–151	GLSKAKEG-VVA *	58
Chain A, Stilbene synthase,	AGLKTTGEGLDWGVLF	357–372	AG-KTK-E----GVLY	50
Chain A, PR 10 protein,	VVGGVALPPT-AEKITFETK	83–101	VVHGVA---TVAEK----TK *	55
Chain A, Protein (peanut lectin)	VSGAVVK-VT	156–164	VGGAVVTGVT	70
Chain A, Arachin Arah3 isoform	EQ-----GAIVT	274–280	EQVTNVGGAVVT	50
Chain A, Arachin Arah3 isoform	AVPTGV	137–142	AVVTGV	83
Chain A, Protein (peanut lectin)	AGSIGGGT	98–105	AGSIAAAT	63

* This match occurred in more than one section of the target sequence.

**Table 8:** Potential cross-reactive epitopes between alpha-synuclein proteins, and shrimp and pineapple bromelain proteins.

**Shrimp Protein**	**Shrimp Protein Sequence**	**Mapped** **Start to End**	**Alpha-Synuclein Sequence**	**ID (%)**
Chain A, Triosephosphate isomerase	FMKTGPLSPNTEVVV	28–42	FMK-G-LSKAKEGVV	60
Chain A, Proliferating cell nuclear antigen	TKEGVKFSAA	163–172	TKEGVVHGVA *	60
Chain A, Triosephosphate isomerase	AIGTGKTA	170–177	AVVTGVTA	63
Chain A, Arginine kinase	QDGILE	343–348	QEGILE	83
**Pineapple Bromelain Protein**	**Pineapple Bromelain** **Sequence**	**Mapped** **Start to End**	**Alpha-Synuclein** **Sequence**	**ID (%)**
Three-Dimensional Structure of Pineapple Cystatin	KAKEQVV	85–91	KAKEGVV *	86
Three-Dimensional Structure of Pineapple Cystatin	AEAEAEEEEG	19–28	AEAAGKTKEG	50

* This match occurred in more than one section of the target sequence.

## Data Availability

Data available in a publicly accessible repository.

## Supplementary Materials

The list of the presently used 180 food’s Current Procedural Terminology (CPT) codes, developed by the American Medical Association, is presented in Supplementary Materials 1. The following are available online at https://www.mdpi.com/article/10.3390/cells10051111/s1.

## Abbreviations

Alpha/α synuclein—aSN; Parkinson’s disease—PD; blood-brain barrier—BBB; wheat germ agglutinin—WGA; post-translational modification of proteins-PTMP; optic density-OD.
