# Supplementary material for: Cross-Reactivity and Sequence Homology Between Alpha-Synuclein and Food Products: A Step Further for Parkinson’s Disease Synucleinopathy
Source: Cells. 2021 May 5;10(5):1111. doi: 10.3390/cells10051111 (PMC8147930; doi:10.3390/cells10051111)
Supplement: Supplementary file 1 [file cells-10-01111-s001.zip › cells-1182069-supplementary.pdf]

The list of the 180 food's CPT codes:

Egg White cooked (CPT CODE : 86256-59)

Egg Yolk cooked (CPT CODE : 86256-59)

Goat`s Milk (CPT CODE : 86256-59)

Soft Cheese + Hard Cheese (CPT CODE : 86256-59)

Yogurt (CPT CODE : 86256-59)

Rice white + brown cooked (CPT CODE : 86256-59)

Rice Cake (CPT CODE : 86256-59)

Rice Protein (CPT CODE : 86256-59)

Rice Endochitinase (CPT CODE : 86256-59)

Wild Rice cooked (CPT CODE : 86256-59)

Wheat + Alpha-Gliadins (CPT CODE : 86256-59)

Black Bean cooked (CPT CODE : 86256-59)

Bean Agglutinins (CPT CODE : 86256-59)

Dark Chocolate + Cocoa (CPT CODE : 86256-59)

Fava Bean cooked (CPT CODE : 86256-59)

Garbanzo Bean cooked (CPT CODE : 86256-59)

Kidney Bean cooked (CPT CODE : 86256-59)

Lentil cooked (CPT CODE : 86256-59)

Lentil Lectin (CPT CODE : 86256-59)

Lima Bean cooked (CPT CODE : 86256-59)

Pinto Beans cooked (CPT CODE : 86256-59)

Soy Sauce gluten-free (CPT CODE : 86256-59)

Soybean Agglutinin (CPT CODE : 86256-59)

Soybean Oleosin + Aquaporin (CPT CODE : 86256-59)

Tofu (CPT CODE : 86256-59)

Almond (CPT CODE : 86256-59)

Almond roasted (CPT CODE : 86256-59)

Brazil Nut raw + roasted (CPT CODE : 86256-59)

Cashew (CPT CODE : 86256-59)

Cashew roasted (CPT CODE : 86256-59)

Cashew Vicilin (CPT CODE : 86256-59)

Chia Seed (CPT CODE : 86256-59)

Flax Seed (CPT CODE : 86256-59)

Hazelnut raw + roasted (CPT CODE : 86256-59)

Macadamia Nut raw + roasted (CPT CODE : 86256-59)

Mustard Seed (CPT CODE : 86256-59)

Peanut roasted (CPT CODE : 86256-59)

Pecan raw + roasted (CPT CODE : 86256-59)

Peanut Butter (CPT CODE : 86256-59)

Peanut Agglutinin (CPT CODE : 86256-59)

Peanut Oleosin (CPT CODE : 86256-59)

Pistachio raw + roasted (CPT CODE : 86256-59)

Pumpkin Seeds roasted (CPT CODE : 86256-59)

Sesame Oleosin (CPT CODE : 86256-59)

Sunflower Seeds roasted (CPT CODE : 86256-59)

Sesame Albumin (CPT CODE : 86256-59)

Walnut (CPT CODE : 86256-59)

Artichoke cooked (CPT CODE : 86256-59)

Asparagus cooked (CPT CODE : 86256-59)

Asparagus (CPT CODE : 86256-59)

Beet cooked (CPT CODE : 86256-59)

Bell Pepper (CPT CODE : 86256-59)

Broccoli (CPT CODE : 86256-59)

Broccoli cooked (CPT CODE : 86256-59)

Brussels Sprouts cooked (CPT CODE : 86256-59)

Cabbage red + green (CPT CODE : 86256-59)

Canola Oleosin (CPT CODE : 86256-59)

Carrot (CPT CODE : 86256-59)

Cauliflower cooked (CPT CODE : 86256-59)

Celery (CPT CODE : 86256-59)

Chili Pepper (CPT CODE : 86256-59)

Cabbage red + green cooked (CPT CODE : 86256-59)

Carrot cooked (CPT CODE : 86256-59)

Corn + Aquaporin cooked (CPT CODE : 86256-59)

Corn Oleosin (CPT CODE : 86256-59)

Cucumber pickled (CPT CODE : 86256-59)

Eggplant cooked (CPT CODE : 86256-59)

Garlic (CPT CODE : 86256-59)

Green Bean cooked (CPT CODE : 86256-59)

Garlic cooked (CPT CODE : 86256-59)

Lettuce(CPT CODE : 86256-59)

Mushroom raw+ cooked (CPT CODE : 86256-59)

Onion+ Scallion (CPT CODE : 86256-59)

Okra cooked (CPT CODE : 86256-59)

Okra cooked (CPT CODE : 86256-59)

Olive green + black pickled (CPT CODE : 86256-59)

Onion + Scallion cooked (CPT CODE : 86256-59)

Pea cooked (CPT CODE : 86256-59)

Pea Protein (CPT CODE : 86256-59)

Pea Lectin (CPT CODE : 86256-59)

Potato white cooked (fried) (CPT CODE : 86256-59)

Potato white cooked (cooked) (CPT CODE : 86256-59)

Popped Corn (CPT CODE : 86256-59)

Pumpkin+ Squash cooked (CPT CODE : 86256-59)

Radish (CPT CODE : 86256-59)

Spinach+ Aquaporin (CPT CODE : 86256-59)

Safflower + Sunflower Oleosin (CPT CODE : 86256-59)

Seaweed (CPT CODE : 86256-59)

Tomato + Aquaporin (CPT CODE : 86256-59)

Tomato Paste (CPT CODE : 86256-59)

Yam+ Sweet Potato cooked (CPT CODE : 86256-59)

Zucchini cooked (CPT CODE : 86256-59)

Apple (CPT CODE : 86256-59)

Apple Cider (CPT CODE : 86256-59)

Apricot (CPT CODE : 86256-59)

Avocado (CPT CODE : 86256-59)

Banana (CPT CODE : 86256-59)

Banana cooked (CPT CODE : 86256-59)

Blueberry (CPT CODE : 86256-59)

Cantaloupe + Honeydew Melon (CPT CODE : 86256-59)

Cherry (CPT CODE : 86256-59)

Cranberry (CPT CODE : 86256-59)

Coconut meat + water (CPT CODE : 86256-59)

Date (CPT CODE : 86256-59)

Fig (CPT CODE : 86256-59)

Grape red + green (CPT CODE : 86256-59)

Grapefruit (CPT CODE : 86256-59)

Kiwi (CPT CODE : 86256-59)

Lemon + Lime (CPT CODE : 86256-59)

Latex Hevein (CPT CODE : 86256-59)

Mango (CPT CODE : 86256-59)

Orange Juice (CPT CODE : 86256-59)

Orange (CPT CODE : 86256-59)

Peach + Nectarine (CPT CODE : 86256-59)

Pear (CPT CODE : 86256-59)

Pineapple (CPT CODE : 86256-59)

Papaya(CPT CODE : 86256-59)

Pineapple Bromelain (CPT CODE : 86256-59)

Plum (CPT CODE : 86256-59)

Pomegranate (CPT CODE : 86256-59)

Red Wine (CPT CODE : 86256-59)

Strawberry (CPT CODE : 86256-59)

Watermelon (CPT CODE : 86256-59)

White Wine (CPT CODE : 86256-59)

Cod cooked (CPT CODE : 86256-59)

Crab + Lobster cooked (CPT CODE : 86256-59)

Clam cooked (CPT CODE : 86256-59)

Halibut cooked (CPT CODE : 86256-59)

Imitation Crab cooked (CPT CODE : 86256-59)

Mackerel cooked (CPT CODE : 86256-59)

Oyster cooked(CPT CODE : 86256-59)

Parvalbumin (CPT CODE : 86256-59)

Red Snapper cooked (CPT CODE : 86256-59)

Salmon (CPT CODE : 86256-59)

Salmon cooked (CPT CODE : 86256-59)

Sardine + Anchovy cooked (CPT CODE : 86256-59)

Sea Bass cooked (CPT CODE : 86256-59)

Shrimp cooked (CPT CODE : 86256-59)

Shrimp Tropomyosin (CPT CODE : 86256-59)

Scallops cooked (CPT CODE : 86256-59)

Squid (Calamari) cooked (CPT CODE : 86256-59)

Tuna cooked (CPT CODE : 86256-59)

Tilapia cooked(CPT CODE : 86256-59)

Trout cooked (CPT CODE : 86256-59)

Tuna raw (CPT CODE : 86256-59)

Whitefish cooked (CPT CODE : 86256-59)

Beef cooked medium (CPT CODE : 86256-59)

Chicken cooked (CPT CODE : 86256-59)

Gelatin(CPT CODE : 86256-59)

Lamb cooked (CPT CODE : 86256-59)

Meat Glue (CPT CODE : 86256-59)

Turkey cooked (CPT CODE : 86256-59)

Pork cooked (CPT CODE : 86256-59)

Basil (CPT CODE : 86256-59)

Cilantro (CPT CODE : 86256-59)

Cumin (CPT CODE : 86256-59)

Dill (CPT CODE : 86256-59)

Mint (CPT CODE : 86256-59)

Oregano (CPT CODE : 86256-59)

Parsley(CPT CODE : 86256-59)

Rosemary (CPT CODE : 86256-59)

Thyme (CPT CODE : 86256-59)

Cinnamon (CPT CODE : 86256-59)

Ginger (CPT CODE : 86256-59)

Clove (CPT CODE : 86256-59)

Nutmeg (CPT CODE : 86256-59)

Paprika (CPT CODE : 86256-59)

Turmeric (Curcumin) (CPT CODE : 86256-59)

Vanilla (CPT CODE : 86256-59)

Carrageenan (CPT CODE : 86256-59)

Beta-Glucan (CPT CODE : 86256-59)

Gum Guar (CPT CODE : 86256-59)

Gum Tragacanth (CPT CODE : 86256-59)

Mastic Gum + Gum Arabic (CPT CODE : 86256-59)

Locust Bean Gum (CPT CODE : 86256-59)

Xanthan Gum (CPT CODE : 86256-59)

Black Tea brewed (CPT CODE : 86256-59)

Coffee Bean Protein brewed (CPT CODE : 86256-59)

Green Tea brewed (CPT CODE : 86256-59)

Food Coloring (CPT CODE : 86256-59)

Honey raw + processed (CPT CODE : 86256-59)
